# MiR-146a rs2910164 (G/C) polymorphism is associated with the development and prognosis of acute coronary syndromes: an observational study including case control and validation cohort

**DOI:** 10.1186/s12967-023-04140-4

**Published:** 2023-05-15

**Authors:** Xiang-Rui Qiao, Tao Zheng, Yifei Xie, Xinyi yao, Zuyi Yuan, Yue Wu, Dong Zhou, Tao Chen

**Affiliations:** 1 Department of Cardiovascular Medicine, The First Affiliated Hospital of Xi’an Jiaotong University, 277 West Yanta Road, Xi’an, Shaanxi 710061 China; 2 Key Laboratory of Molecular Cardiology, Key Laboratory of Environment and Genes Related to Diseases, Ministry of Education, Xi’an Jiaotong University, 277 West Yanta Road, Xi’an, Shaanxi 710061, China; 3 Department of Cardiovascular Medicine, Yongchuan Hospital of Chongqing Medical University, 439 XuanHua Road, Chongqing, 402160 China

**Keywords:** Acute coronary syndrome, MicroRNA, Single nucleotide polymorphism, Inflammation

## Abstract

**Background:**

Polymorphisms in microRNAs (miRNAs) play an important role in acute coronary syndromes (ACS). The purpose of this study was to assess the association of miR-146a rs2910164 and miR-34b rs4938723 polymorphisms with the development and prognosis of ACS and to explore the underlying mechanisms.

**Methods:**

A case–control study of 1171 subjects was included to determine the association of miR-146a rs2910164 and miR-34b rs4938723 polymorphisms with ACS risk. An additional 612 patients with different miR-146a rs2910164 genotypes, who underwent percutaneous coronary intervention (PCI) were included in the validation cohort and followed for 14 to 60 months. The endpoint was major adverse cardiovascular events (MACE). A luciferase reporter gene assay was used to validate the interaction of oxi-miR-146a(G) with the IKBA 3'UTR. Potential mechanisms were validated using immunoblotting and immunostaining.

**Results:**

The miR-146a rs2910164 polymorphism was significantly associated with the risk of ACS (Dominant model: CG + GG vs. CC, OR = 1.270, 95% CI (1.000–1.613), P = 0.049; Recessive model: GG vs. CC + CG, OR = 1.402, 95% CI (1.017–1.934), P = 0.039). Serum inflammatory factor levels were higher in patients with the miR-146a rs2910164 G allele than in those with the C allele. MiR-146a rs2910164 polymorphism in dominant model was associated with the incidence of MACE in post-PCI patients (CG + GG vs. CC, HR = 1.405, 95% CI (1.018–1.939), P = 0.038). However, the miR-34b rs4938723 polymorphism was not associated with the prevalence and prognosis of ACS. The G allele of miR-146a rs2910164 tends to be oxidized in ACS patients. The miRNA fractions purified from monocytes isolated from ACS patients were recognized by the 8OHG antibody. Mispairing of Oxi-miR-146a(G) with the 3'UTR of IKBA results in decreased IκBα protein expression and activation of the NF-κB inflammatory pathway. P65 expression was higher in atherosclerotic plaques from patients carrying the miR-146a rs2910164 G allele.

**Conclusion:**

The variant of miR-146a rs2910164 is closely associated with the risk of ACS in Chinese Han population. Patients carrying miR-146a rs2910164 G allele may have worse pathological change and poorer post-PCI prognosis, partly due to the oxidatively modified miR-146a mispairing with 3′UTR of IKBA and activating NF-κB inflammatory pathways.

**Supplementary Information:**

The online version contains supplementary material available at 10.1186/s12967-023-04140-4.

## Introduction

Despite significant advances in the diagnosis and treatment of coronary artery disease (CAD), cardiovascular disease remains the leading cause of death worldwide, with nearly half of these deaths due to ischemic heart disease [1, 2]. As the most important type of ischemic heart diseases, acute coronary syndromes (ACS), characterized pathologically by unstable atherosclerotic lesion, commonly begin with atherosclerotic plaque rupture and intracoronary thrombus formation and result in cardiovascular disease events [3].

MicroRNAs (miRNAs) are approximately 22 nucleotide RNAs that negatively regulate gene expression by inhibiting mRNA translation or promoting mRNA degradation [4] thereby participate in physiological processes such as cell differentiation and proliferation, apoptosis, and organ development [5, 6]. It has been demonstrated that miRNAs are closely related to the pathogenesis of cardiovascular disease [7]. MiR-146a and miR-34b are significantly up-regulated in human atherosclerotic plaques [8]. In addition, circulating miR-146a levels were significantly increased in ACS patients [9]. Furthermore, the inhibition of the miR-34 family reduces ventricular remodeling and cardiac dysfunction due to myocardial infarction (MI) or pressure overload, and increases angiogenesis [10].

The polymorphism of miRNAs may alter their target selection and change their original biological function, leading to gene susceptibility as one of the risk factors in many diseases [11]. Polymorphisms of miR-146a rs2910164 and miR-34b rs4938723 have been reported to be associated with the development and prognosis of Crohn’s disease and cancer [12, 13]. Recent studies have further demonstrated that miR-146a rs2910164 is associated with the pathogenesis of ischemic stroke [14]. However, no studies have elaborated the roles of miR-146a rs2910164 and miR-34b rs4938723 in the development of ACS and their underlying mechanisms.

Our present work demonstrated that the miR-146a rs2910164, not miR-34b rs4938723, polymorphism is a risk factor for ACS and that patients carrying the G allele have higher mortality after percutaneous coronary intervention (PCI). Our data suggest a mechanism by which oxidatively modified miR-146a rs2910164 mismatches with the 3ʹUTR of IKBA, thereby activating NF-κB inflammatory pathways that leads to cardiovascular events.

## Methods

### Study population

This research consists of two independent studies: a case–control and a validation cohort. In the case–control study, we screened a total of 1605 CAD patients and finally included 1171 Han patients aged 24–80 years. Patients with stable angina (SA) were included in the control group (n = 552). The ACS group (n = 619) included patients with acute ST-segment elevation myocardial infarction (STEMI), acute non-ST-segment elevation myocardial infarction (NSTEMI), and unstable angina (UA).

The prospective cohort study screened a total of 697 patients with ACS undergoing primary percutaneous coronary intervention (PCI), 53 patients were excluded according to the exclusion criteria, 32 patients (4.5%) were lost to follow-up, and finally 612 patients were included in the study. Based on the genetic analysis, they were divided into miR-146a rs2910164 CC, CG and GG groups. Subjects with different genotypes were followed for 14 to 60 months (median follow-up time is 42 months) to assess the incidence of mortality and MACE. The endpoint was major adverse cardiovascular events (MACE), including all-cause death, nonfatal acute myocardial infarction, revascularization, and stroke.

This study was conducted at the First Affiliated Hospital of Xi'an Jiaotong University from January 2016 to October 2021. Patients were excluded from the case–control study and the cohort study with the following cases: acute infections, post-revascularization, serious liver or kidney disease, cancer or acute stroke. Written informed consent was obtained from all study participants, and this study was approved by the ethnic committee approval of the First Affiliated Hospital of Xi’an Jiaotong University.

### Clinical measurements

Well-trained interviewers and investigators gathered all samples and clinical data. A history of smoking was defined as either past or current history of cigarettes. Hypertension was defined as either the systolic blood pressure higher than 140 mmHg or diastolic blood pressure higher than 90 mmHg. Diabetes mellitus (DM) was defined as fasting plasma glucose ≥ 7.0 mmol/L or use of anti-diabetic therapies, or HbA1c ≥ 6.5%. The body weight (kg) and height (m) of each subject were acquired during the initial visit. Body mass index (BMI) was calculated by the equation of body weight (kg)/height square (m^2^).

### Genetic analysis

Genomic DNA was extracted from white blood cells using the commercially available DNA isolation kit (Tiangen Biotech, Beijing, China) according to the manufacturer’s instructions. Genotypes for individual DNA samples were genotyped using the ABI PRISM- Snapshot method (Applied Bio system, CA, USA). More detail information was shown in Additional files. Genotypes of 2 SNPs were identified by capillary electrophoresis (ABI PRISM3730 DNA Sequencer; Applied Biosystems). The results were analyzed with GeneMapper 3.0 software (Applied Biosystems). All SNapShot and PCR primers were listed in Additional file 1: Table S2. Genotyping quality control procedures for study have been described in detail [15]. In order to evaluate the multiplex SNaPShot results, 40 samples were randomly selected and regenotyped by direct sequencing using a BigDye terminator (Applied Biosystem). All assays were 100% concordant.

### Measurement of serum cytokines

Overnight after admission, all blood samples (5 mL) were collected into tubes containing anticoagulant and then were centrifuged for 10 min at 3000 rpm. The concentrations of serum high sensitivity C-reactive protein (hs-CRP), fasting glucose, high density lipoprotein cholesterol(HDL-C) and triglyceride(TG) were determined at the laboratory in the hospital. Low-density lipoprotein cholesterol (LDL-C) was calculated using the Friedewald formula. Interleukin-1β (IL-1β), interleukin-6 (IL-6), and tumor necrosis factor-α (TNF-α) in serum were determined by ELISA kits (R&D Systems, Minneapolis, MN, USA).

### Calculation of Gensini’s score

The Gensini score was calculated for each patient from the coronary angiogram. Gensini score grades a severity score to each coronary stenosis as 1 for 1 to 25% narrowing, 2 for 26 to 50%, 4 for 51 to 75%, 8 for 76% to 90%, 16 for 91% to 99%, and 32 for a completely occluded artery. The score is then multiplied by a factor according to the importance of the coronary artery. The multiplication factor is 5 for a left main coronary artery, 2.5 for proximal left anterior descending artery and proximal circumflex artery, 1.5 for a mid left anterior descending artery, and 1 for distal left anterior descending artery, mid or distal circumflex artery, and right coronary artery. The multiplication factor for any other branch is 0.5. Gensini score was showed as the sum of the scores for all coronary arteries.

### Statistical analysis

Categorical variables are presented (%) and continuous variables are presented as mean ± SD. Data from all groups were compared using theχ2 test for categorical variables and a Student t test for continuous variables. The odds ratio and 95% confidence intervals (CI) were calculated using logistic regression to achieve the relative risks of various genotypes for ACS. The association between different genotypes and the risks of MACE was estimated by Kaplan–Meier survival and Cox regression analysis. Hazard ratios (HR) were presented with 95% CI to reveal the risk of an event when this factor is appeared. All calculations and graphs were performed using R version 4.2.2 (Vienna, Austria) and Graphpad prism version 8.0 softwares. Statistically significant differences were defined as P < 0.05 using a two-tailed test.

## Results

### MiR-146a and miR-34b, two microRNAs with rs2910164 and rs4938723 polymorphisms, are highly expressed in CAD patients

**Fig. 1:**
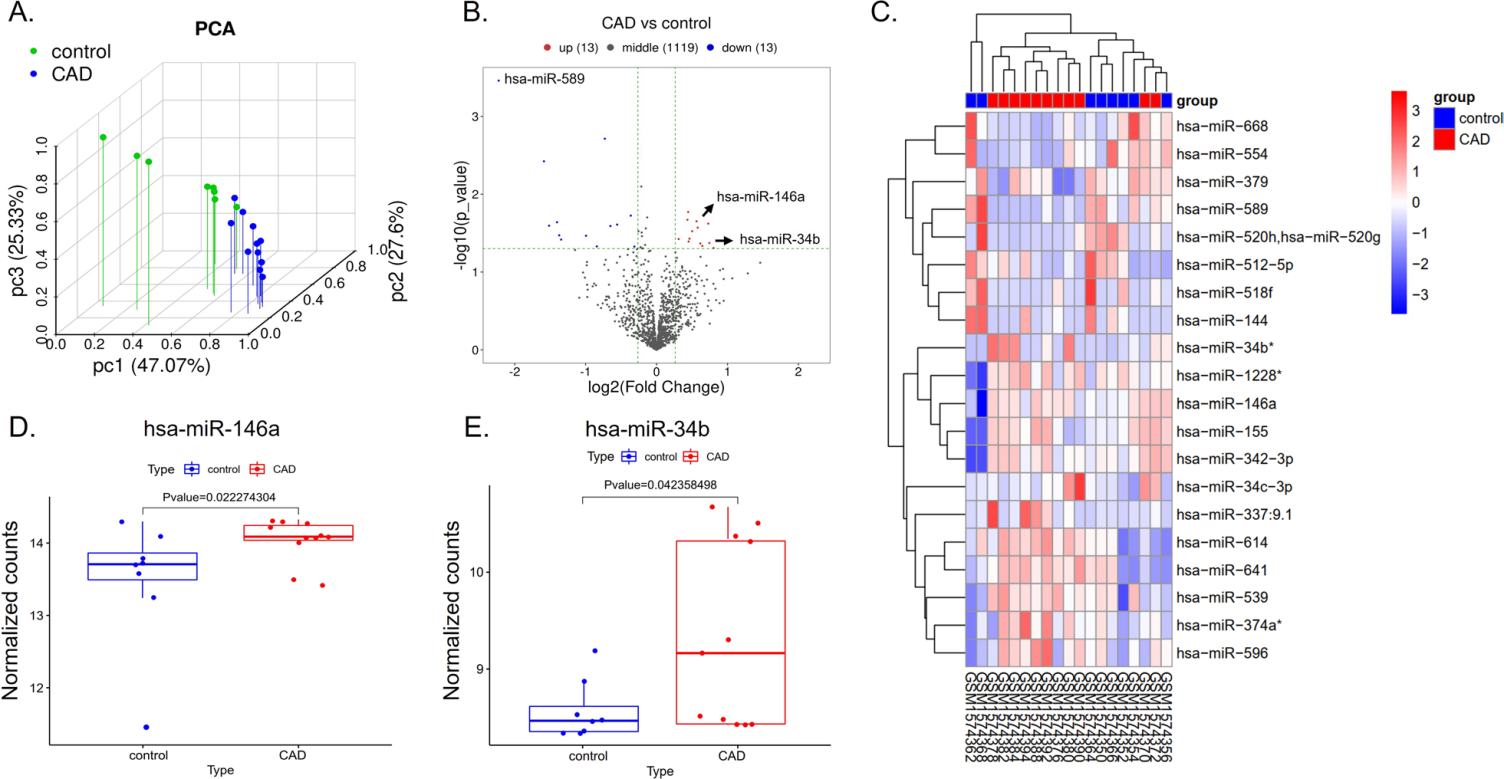
MiR-146a and miR-34b are highly expressed in CAD patients. **A** Principal component analysis (PCA) of GSE64566 dataset from the NCBI_GEO database (https://www.ncbi.nlm.nih.gov/gds/); **B** Volcano plot. Red represents up-regulated genes; blue represents down-regulated genes; black represents genes with no significant difference in expression, each point in the figure represents a gene, and the screened differential genes are genes within the range of p-value < 0.05, |FC|> 1.2; **C** Heatmap. Each small square in the figure represents 1 gene in 1 sample, and its color represents the expression value of the gene. The darker the color, the higher the gene expression level (red for up-regulated genes, blue for down-regulated genes). Differentially expressed genes with p-value < 0.05 and |FC|≥ 1.2 are shown in the figure; **D**, **E**. Boxplot of miR-146a and miR-34b expression

To determine which microRNAs are highly expressed in patients with coronary artery disease (CAD), we downloaded the non-coding RNA dataset GSE64566 from the NCBI_GEO database (https://www.ncbi.nlm.nih.gov/gds) [16]. Epicardial adipose tissues (EAT) from control (n = 8) and CAD (n = 11) patients was used as samples for differential gene expression analysis. Principal component analysis (PCA) revealed significant differences in gene expression between the control and CAD groups (Fig. 1A). Volcano plots and heatmaps showed increased expression of 13 microRNAs in CAD patients (Fig. 1B, C). See Additional file 2: Excel1_Different miRNA for details. We then performed a secondary analysis of the data on microRNA expression in atherosclerotic plaques provided by Emma Raitoharju’s article[8] and found that seven microRNAs were highly expressed in atherosclerotic plaques (Additional file 1: Table S1). By comparison, we found that miR-146a and miR-34b were increased in both epicardial fat and atherosclerotic plaques in CAD patients (Fig. 1C–E, Additional file 1: Table S1). Next, All SNPs of miR-146a and miR-34b were then screened using the online tool miRNASNP V3 (http://bioinfo.life.hust.edu.cn/miRNASNP#!/). We excluded SNPs with minor allele frequency (MAF) < 0.05 and finally targeted two SNPs: miR-146a rs2910164 and miR-34b rs4938723. Several literatures [14, 17, 18] have reported that miR-146a rs2910164 and miR-34b rs4938723 polymorphisms are associated with CAD and ischemic stroke. However, there are few reports on the association of the two SNPs with acute coronary syndrome (ACS). Therefore, we hypothesize that miR-146a rs2910164 and miR-34b rs4938723 may also be involved in the development of ACS.

### MiR-146a rs2910164, but not miR-34b rs4938723, polymorphism is a risk factor for ACS

**Fig. 2:**
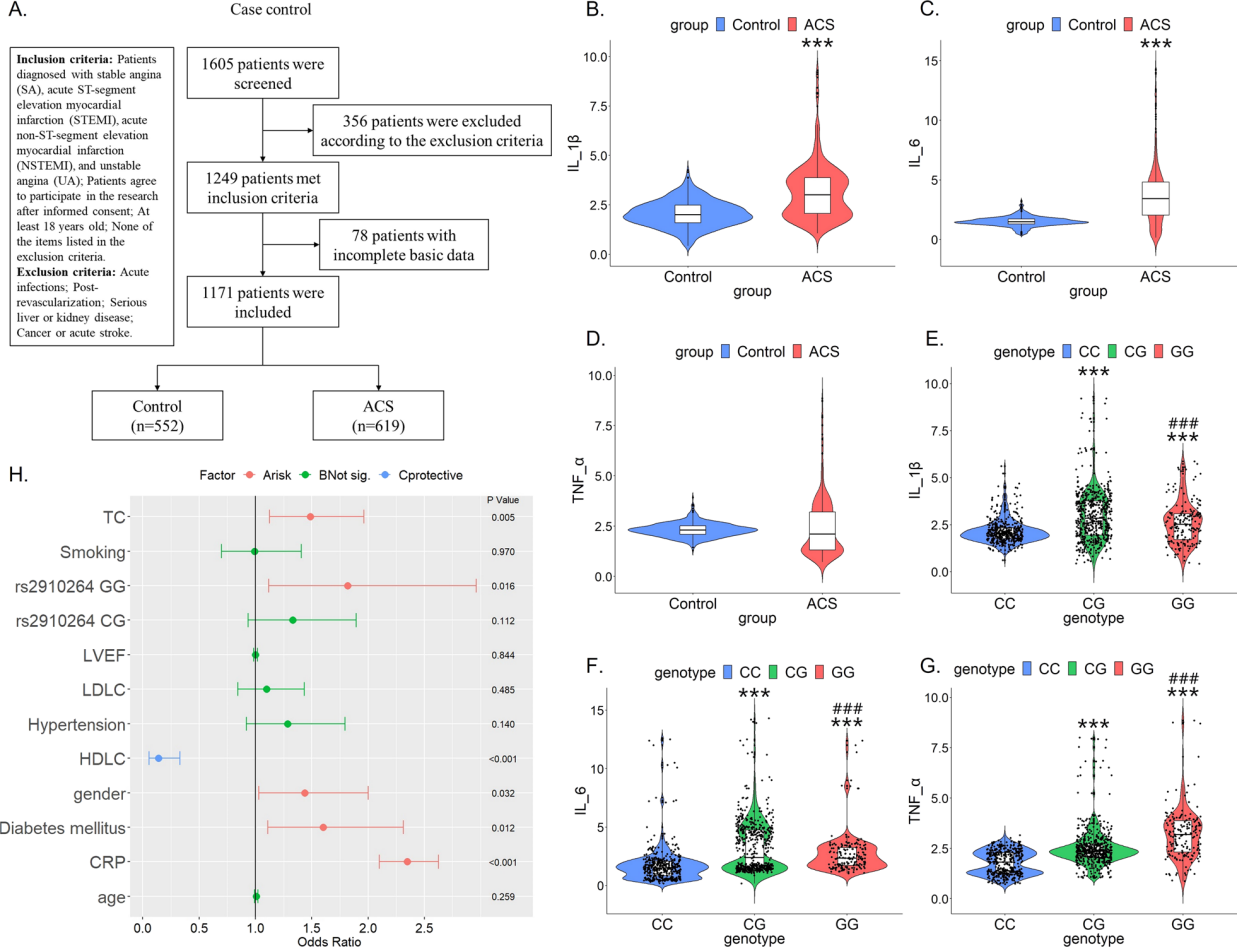
MiR-146a rs2910164 polymorphism is a risk factor for ACS. **A** Flow chart of case control study; **B**–**D** Serum IL-1β, IL-6 and TNF-α levels in control group and ACS group. The unit is pg/ml. ***p < 0.001 versus Control; **E**–**G** Among all 1171 individuals, serum IL-1β, IL-6 and TNF-α levels in different genotypes of miR-146a rs2910164. The unit is pg/ml. ***p < 0.001 versus CC genotype, ### p < 0.001 versus CG genotype; **H** Forest plot

We screened 1605 CAD patients and eventually identified 1171 patients (Fig. 2A). Among them, patients with stable angina pectoris (SA) were included in the control group (n = 552). Patients with acute ST-segment elevation myocardial infarction (STEMI), acute non-ST-segment elevation myocardial infarction (NSTEMI), and unstable angina pectoris (UA) were included in the ASC group (n = 619). Comparing baseline data between the two groups, we found that the ACS group was older, had higher rates of smoking, hypertension, and family history, and had worse cardiac and renal function (Table 1). Furthermore, serum levels of inflammatory factors IL-1β and IL-6 were significantly higher in the ACS group than in the control group (p < 0.001). Although TNF-α levels were also higher in the ACS group than in the control group, there was no statistical difference. (Fig. 2B–D).

**Table 1:** Basic information of control subjects and ACS patients

	Control (n = 552)	ACS (n = 619)	P value
Age, y	56.67 ± 10.87	58.45 ± 10.96	0.006
Male, n (%)	272 (49.3)	340 (54.9)	0.053
Smoking, n (%)	170(30.8)	225(36.3)	0.045
Drinking, n (%)	112(20.3)	123(19.9)	0.858
Hypertension, n (%)	270(48.9)	342(55.3)	0.030
Diabetes mellitus, n (%)	124(22.5)	168(27.1)	0.065
Family history, n (%)	90(16.3)	134(21.5)	0.020
BMI, kg/m^2^	24.80 ± 3.43	25.16 ± 3.10	0.063
SBP, mmHg	126.82 ± 19.60	124.74 ± 21.57	0.084
DBP, mmHg	79.28 ± 13.00	77.98 ± 13.56	0.096
Heart rate, bmp	73.63 ± 10.14	74.83 ± 15.04	0.107
LVEF, %	56.60 ± 8.88	55.25 ± 11.04	0.021
Creatinine, mg/dl	79.09 ± 16.48	81.42 ± 22.06	0.042
FBG, mmol/l	6.42 ± 1.45	6.72 ± 1.91	0.002
CK, U/l(IQR)	73.65(58.88)	91.50(80.20)	< 0.001
CK-MB, U/l(IQR)	11.60(5.58)	13.50(10.50)	< 0.001
FIB, g/l	3.05 ± 1.15	3.72 ± 1.14	< 0.001
D-Dimer, mg/l(IQR)	0.56(0.84)	0.85(0.70)	< 0.001
FDP, mg/l	3.04 ± 1.15	3.72 ± 1.72	< 0.001
CRP, μg/ml	2.75 ± 0.92	6.47 ± 2.94	< 0.001
BNP, ng/l(IQR)	124.99(193.56)	418.87(961.20)	< 0.001
TC, mmol/l	3.82 ± 0.77	4.01 ± 1.07	< 0.001
TG, mmol/l	1.87 ± 0.86	1.93 ± 0.80	0.282
HDLC, mmol/l	1.01 ± 0.16	0.99 ± 0.23	0.030
LDLC, mmol/l	2.24 ± 0.77	2.38 ± 0.91	0.004
ApoA1, g/l	1.01 ± 0.20	1.02 ± 0.20	0.425
ApoB, g/l	0.76 ± 0.24	0.79 ± 0.24	0.113
ApoE, g/l	35.89 ± 12.57	36.85 ± 14.40	0.230
Lpa, mg/dl	196.32 ± 69.37	207.00 ± 99.72	0.032
Hb, g/l	136.25 ± 16.81	138.27 ± 17.39	0.044
WBC, 10^9^ /l	6.01 ± 2.47	9.00 ± 4.43	< 0.001
ACEI, %	308(55.8)	364(58.8)	0.299
β-blocker, %	430(77.9)	509(82.2)	0.063
Statin, %	476(86.2)	555(89.7)	0.071
Aspirin, %	519(94.0)	595(96.1)	0.095
CCB, %	153(27.7)	407(65.8)	< 0.001

*BMI* body mass index; *SBP* systolic blood pressure; *DBP* diastolic blood pressure; *LVEF* left ventricular ejection fraction; *FBG* fasting blood glucose; *CK* creatine kinase; *CK-MB* creatine kinase isoenzymes; *FIB* fibrinogen; *FDP* fructose-1, 6-diphosphate; *CRP* C-reactive protein; *BNP* brain natriuretic peptide; *TC* total cholesterol; TG: triglyceride; *HDL* high-density lipoprotein cholesterol; *LDL* low-density lipoprotein cholesterol; *Apo* Apolipoprotein; *Lpa* lipoprotein a; *HGB* hemoglobin; *WBC* white blood cell count; *ACEI* angiotensin converting enzyme inhibitor; *ARB* angiotensin receptor blocker; *CCB* calcium channel blocker

The Hardy–Weinberg law of equilibrium (HWE) is the most important principle of population inheritance. In Table 2, after the HWE test was performed on both the control and the ACS group, it was confirmed that the gene distribution of miR-146a rs2910164 and miR-34b rs4938723 loci in the two groups were in line with the Hardy–Weinberg equilibrium (rs2910164: p of control group = 0.614, p of ACS group = 0.975; rs4938723: p of control group = 0.478, p of ACS group = 0.111). Subsequently, we performed a chi-square test on the miR-146a rs2910164 and miR-34b rs4938723 SNPs between the two groups and found that the miR-146a rs2910164 SNP was a risk factor for ACS in both dominant and recessive modes (Dominant model: OR = 1.270, 95%CI (1.000, 1.613), p = 0.049; Recessive model: OR = 1.402, 95%CI (1.017, 1.934), p = 0.039). Whereas, the difference of miR-34b rs4938723 SNP between the two groups was not statistically significant in both dominant and recessive modes (Table 2). In addition, as shown in Fig. 2E–G, among all 1171 patients, serum IL-1β, IL-6 and TNF-α levels were significantly higher in patients carrying the miR-146a rs2910164 G allele (CG + GG genotype) than in those carrying the miR-146a rs2910164 C allele (CC genotype). However, there was no difference in serum inflammatory factor levels among patients with miR-34b rs4938723 TT, TC and CC genotypes (Additional file 1: Figure S1 A-C). Finally, Age, gender, smoking, hypertension, diabetes, LVEF, serum CRP, TC, LDLC and HDLC levels, rs2910164 CG and rs2910164 GG were included to construct a multifactorial logistic regression equation. The results found that: men were more likely to develop ACS than women (OR = 1.438, 95%CI (1.032, 2.004), p = 0.032); diabetes increased the risk of ACS (OR = 1.602, 95%CI (1.109, 2.314), p = 0.012); higher serum CRP and TC levels were associated with a higher risk of ACS (CRP: OR = 2.348, 95%CI (2.010, 2.626), p < 0.001; TC: OR = 1.486, 95%CI (1.125, 1.963), p = 0.005); higher serum HDLC levels were associated with a lower risk of ACS (OR = 0.138, 95%CI (0.058, 0.331), p < 0.001); and most importantly rs2910164 GG remained a risk factor for ACS (OR = 1.820, 95%CI (1.119, 2.961), p = 0.016) (Fig. 2H). These results suggest that miR-146a rs2910164 polymorphism is a risk factor for ACS, and patients carrying miR-146a rs2910164 G allele have higher levels of inflammation.

**Table 2:** Association of miRNA polymorphisms with ACS Risk

	Control (n = 552)	ACS (n = 619)	χ2	OR (95% CI)	P Value
miR146aC > G					
CC	216 (39.13%)	208 (33.60%)		1.000 (reference)	
CG	263 (47.64%)	302 (48.79%)	1.873(vs. CC)	1.192 (0.927–1.534)	0.171
GG	73 (13.23%)	109 (17.61%)	5.991 (vs. CC)	1.551 (1.090–2.205)	0.014
Dominant model (CC vs. CG + GG)			3.860	1.270(1.000–1.613)	0.049
Recessive model (CC + CG vs. GG)			4.273	1.402 (1.017–1.934)	0.039
HWE χ2	0.254	0.001			
HWE *p*	0.614	0.975			
miR34bT > C					
TT	245 (44.38%)	291 (47.01%)		1.000 (reference)	
TC	251 (45.47%)	279 (45.07%)	0.291(vs. TT)	0.936 (0.736 -1.191)	0.589
CC	56 (10.15%)	49 (7.92%)	2.049(vs. TT)	0.737 (0.484 -1.121)	0.152
Dominant model (TT vs. CT + CC)			0.811	0.900 (0.714–1.133)	0.368
Recessive model (TT + CT vs. CC)			1.776	0.761(0.509–1.138)	0.183
HWE χ2	0.503	2.543			
HWE *p*	0.478	0.111			

*miRNA* microRNA; *ACS* acute coronary syndrome; *OR* Odds ratio; *CI* Confidence interval; *HWE* Hardy–Weinberg equilibrium test

### MiR-146a rs2910164 polymorphism is associated with inflammatory factor levels and the severity of coronary lesions in the ACS group

**Fig. 3:**
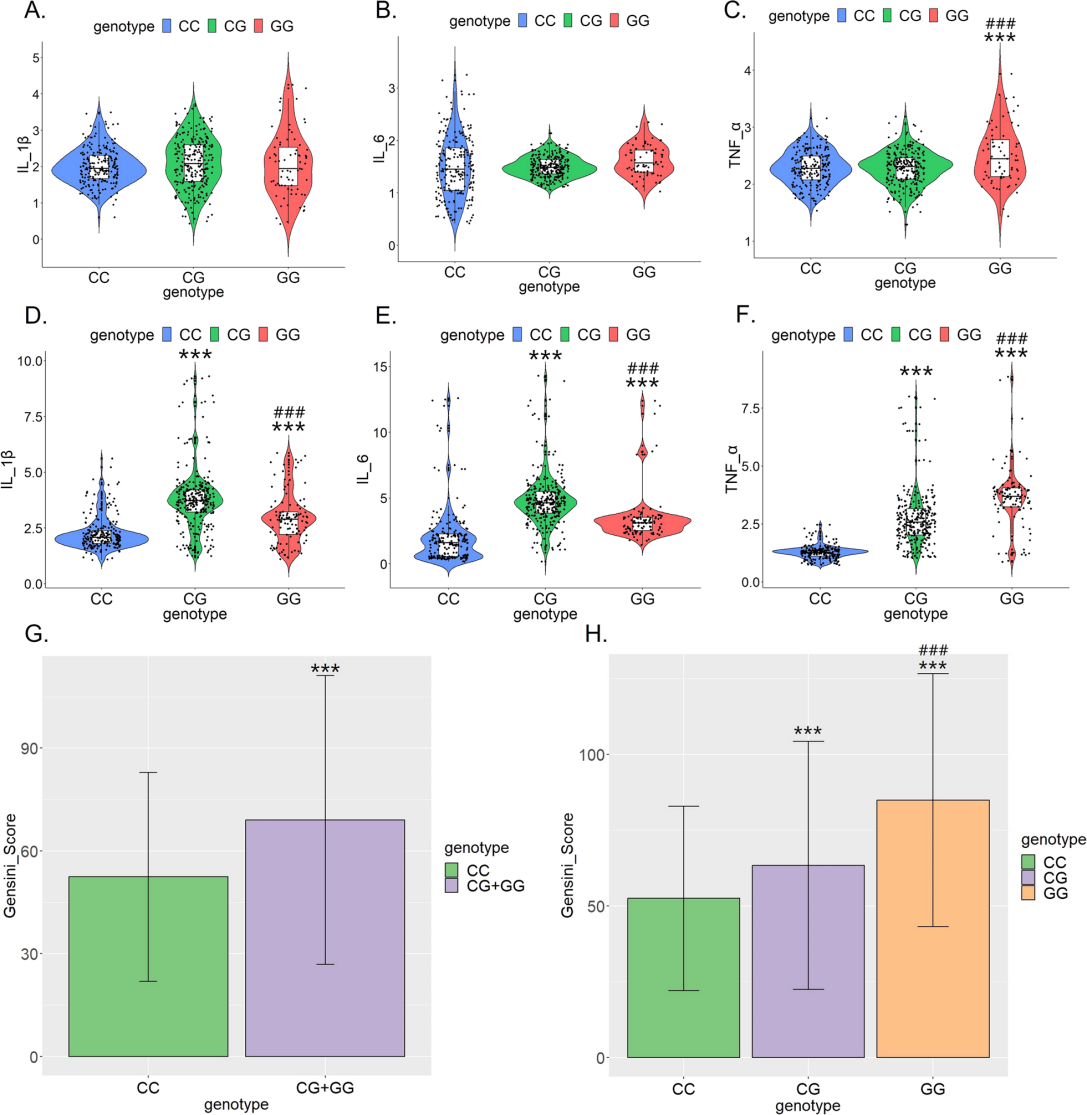
MiR-146a rs2910164 polymorphism is associated with inflammatory factor levels and the severity of coronary lesions in the ACS group. **A**–**C** Within control group, serum IL-1β, IL-6 and TNF-α levels in different genotypes of miR-146a rs2910164. The unit is pg/ml. ***p < 0.001 versus CC genotype, ### p < 0.001 versus CG genotype; **D**, **E** Within ACS group, serum IL-1β, IL-6 and TNF-α levels in different genotypes of miR-146a rs2910164. The unit is pg/ml. ***p < 0.001 versus CC genotype, ### p < 0.001 versus CG genotype; **F**–**G** Within ACS group, Gensini scores of patients with different genotypes of miR-146a rs2910164. **p < 0.01 and ***p < 0.001 versus CC genotype, ### p < 0.001 versus CG genotype

We examined the relationship between the above two SNPs and inflammatory factor levels within the control and ACS groups, respectively, and found that the miR-146a rs2910164 polymorphism was weakly associated with inflammatory factor levels in the control group (Fig. 3A–C). Control individuals with GG genotype had slightly higher TNF-α serum level than CC and CG genotypes. However, IL-1β and IL-6 serum levels did not differ among individuals with the three genotypes (Fig. 3A–C). Surprisingly, within the ACS group, the three serum inflammatory factor levels were significantly higher in patients with the CG and GG genotypes than in patients with the CC genotype. And the TNF-α level of patients with GG genotype was higher than that of CG genotype patients (Fig. 3D–F). The miR-34b rs4938723 polymorphism was not associated with inflammatory factor levels in either the control or ACS groups (Additional file 1: Figure S1D–I).

Gensini score takes full account of the number, location and extent of coronary lesions and is a commonly used and relatively scientific evaluation standard based on angiography to assess the severity of coronary atherosclerosis. Within the ACS group, the mean Gensini scores of patients with miR-146a rs2910164 CC, CG, and GG genotypes were 52.46 ± 2.11, 63.31 ± 2.35, and 84.89 ± 4.00, respectively. As shown in Fig. 3G, H, patients carrying the G allele had significantly higher Gensini scores: the score of patients with CG genotype was significantly higher than that of patients with CC genotype, and it was further increased in patients with GG genotype. Taken together, it appears that patients carrying the G allele had higher levels of inflammation and more severe coronary lesions.

### Polymorphism of miR-146a rs2910164 was associated with the outcome of post-PCI patients

**Fig. 4:**
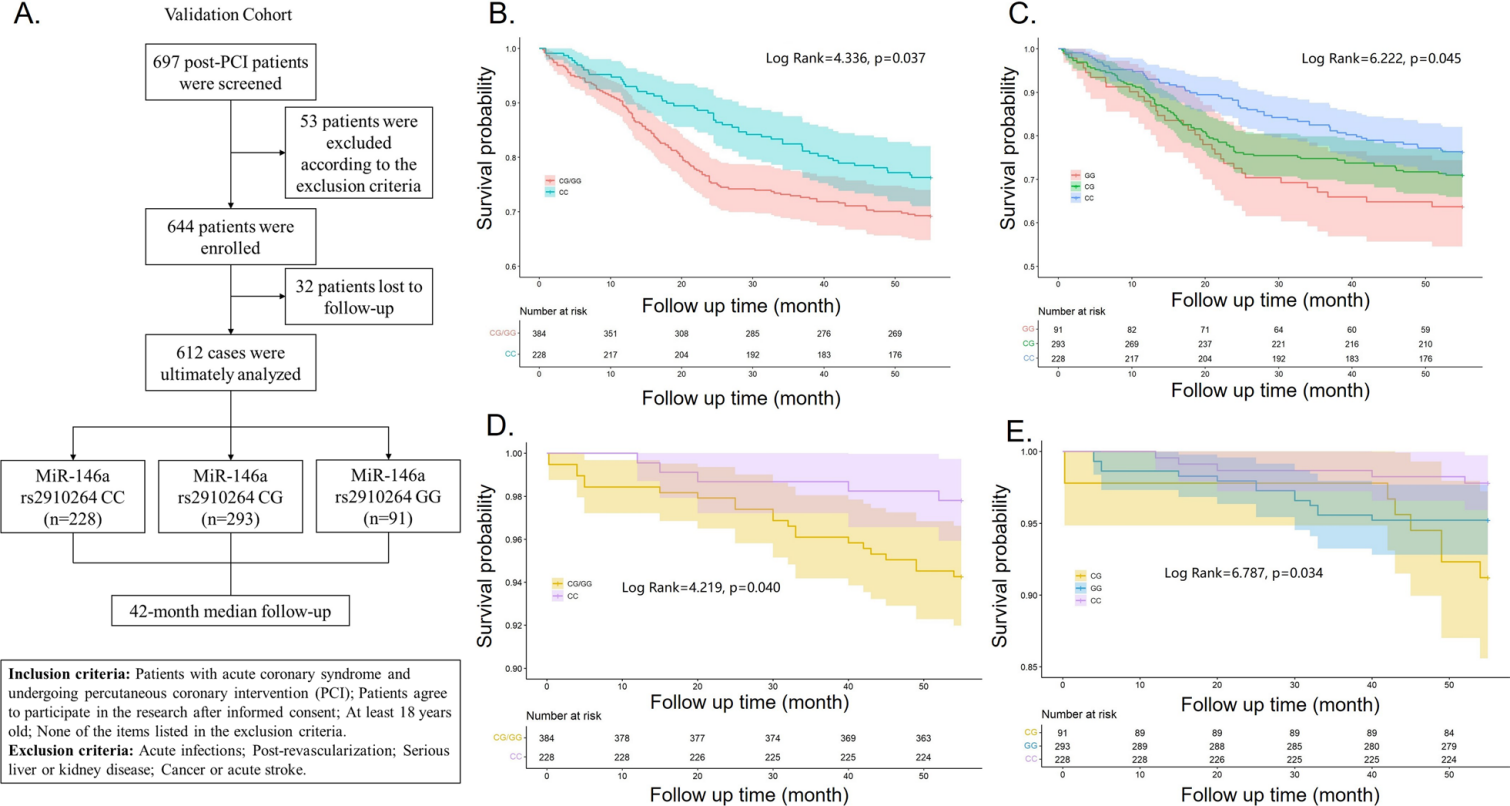
Kaplan–Meier curve of clinical outcomes of post-PCI patients. **A** Flow chart of validation cohort; **B**, **C** Kaplan–Meier survival curve for MACE judging by miR-146a rs2910164 polymorphism. MACE: major adverse cardiovascular events; **D**, **E** Kaplan–Meier survival curve for death judging by miR-146a rs2910164 polymorphism

The above study found that miR-146a rs2910164 polymorphism was associated with levels of inflammatory factors only in the ACS group, and that patients carrying the G allele had more severe coronary lesions, so we hypothesized that it might be related to the prognosis of post-PCI patients. We screened 697 post-PCI patients, and 612 patients were finally included in the validation cohort due to inclusion and exclusion criteria and loss to follow-up (Fig. 4A). They were categorized into CC, CG, and GG groups according to miR-146a rs2910164 genotypes, and were followed up for 14 to 60 months (median follow-up time is 42 months). Baseline data showed that patients with CG genotype were older than patients with CC genotype. GG genotype patients had higher lipoprotein a (Lpa) levels than CC genotype patients, and their fibrinogen (FIB) levels were higher than CG genotype patients (Table 3). The rest of the medical history, biochemical indicators and medication history did not differ significantly among patients with different genotypes (Table 3). Proportional hazards model was used to detect the correlation between miR-146a rs2910164 polymorphism and MACE. Table 4 showed that in the dominant model post-PCI patients with G allele were more prone to have MACE (HR = 1.405 95% CI (1.018–1.939), P = 0.038). Kaplan–Meier survival curves also indicated that patients with the GG genotype were most likely to have MACE compared to those with the CG and CC genotypes (Fig. 4B, C, Additional file 1: Figure S2A, B). In addition, Kaplan–Meier curves for mortality indicated that patients carrying G allele had higher mortality compared with patients carrying C allele (Fig. 4D, E Additional file 1: Figure S2C, D). In brief, these data indicate that miR-146a rs2910164 polymorphism was associated with the outcome of post-PCI patients.

**Table 3:** Basic information of patients with different miR-146a rs2910164 genotypes

	CC (n = 228)	CG (n = 293)	GG (n = 91)
Age, y	57.07 ± 11.41	59.22 ± 10.81*	59.01 ± 9.63
Male, n (%)	192(84.2)	243(82.9)	72(79.1)
Smoking, n (%)	138(60.5)	187(63.8)	60(65.9)
Hypertension, n (%)	107(46.9)	140(47.8)	48(52.7)
Diabetes mellitus, n (%)	43(18.9)	44(15.0)	12(13.2)
Family history, n (%)	13(5.7)	16(5.5)	11(12.1)
SBP, mmHg	123.72 ± 19.19	126.47 ± 23.40	125.85 ± 22.31
DBP, mmHg	78.13 ± 11.96	78.86 ± 14.52	77.34 ± 12.53
Heart rate, bmp	74.19 ± 12.06	75.02 ± 16.41	75.92 ± 14.74
LVEF, %	56.32 ± 11.51	56.84 ± 11.46	56.44 ± 11.04
Creatinine, mg/dl	81.16 ± 20.37	79.12 ± 24.60	78.08 ± 21.11
FBG, mmol/l	7.67 ± 3.46	7.12 ± 3.02	7.82 ± 3.49
UA, μmol/l	315.08 ± 92.79	320.13 ± 91.82	313.51 ± 118.39
FIB, g/l	3.58 ± 1.33	3.53 ± 1.42	3.86 ± 1.46^#^
D-Dimer, mg/l (IQR)	0.80(0.65)	0.79(0.80)	0.85(0.83)
CRP, μg/ml(IQR)	2.28(5.55)	1.82(5.49)	1.90(6.19)
BNP, ng/l(IQR)	275.90(936.23)	163.30(889.97)	164.3 (850.23)
TC, mmol/l	4.01 ± 0.99	4.02 ± 1.05	4.22 ± 1.13
TG, mmol/l	1.68 ± 0.94	1.67 ± 1.13	1.59 ± 1.01
HDLC, mmol/l	1.01 ± 0.24	0.98 ± 0.25	1.02 ± 0.22
LDLC, mmol/l	2.33 ± 0.73	2.38 ± 0.82	2.51 ± 0.95
ApoA1, g/l	1.05 ± 0.20	1.04 ± 0.22	1.65 ± 0.57
ApoB, g/l	0.77 ± 0.23	0.80 ± 0.30	0.83 ± 0.30
Lpa, mg/dl	184.75 ± 176.93	187.86 ± 150.28	225.60 ± 189.41*
ACEI, n (%)	206(90.3)	264(90.1)	84(92.3)
β-blocker, n (%)	201(88.2)	250(85.3)	85(93.4)
Statin, n (%)	217(95.2)	281(95.9)	81(89.0)

*SBP* systolic blood pressure; *DBP* diastolic blood pressure; *LVEF* left ventricular ejection fraction; *FBG* fasting blood glucose; *FIB* fibrinogen; *CRP* C-reactive protein; *BNP* brain natriuretic peptide; *TC* total cholesterol; TG: triglyceride; *HDL* high-density lipoprotein cholesterol; *LDL* low-density lipoprotein cholesterol; *Apo* Apolipoprotein; *Lpa* lipoprotein a; *ACEI* angiotensin converting enzyme inhibitor; *p < 0.05 versus CC genotype, #*p* < 0.05 versus CG genotype

**Table 4:** Association of miR-146a rs2910164 polymorphisms with MACE

	Patients without MACE	Patients with MACE	HR(95%CI)	P value
	(n = 440)	(n = 172)		
miR146aC > G				
CC	160 (36.36%)	47 (27.33%)	1.000 (reference)	
CG	213 (48.41%)	89 (51.74%)	1.146 (0.966–1.359)	0.118
GG	67 (15.23%)	36 (20.93%)	1.732 (1.123–2.671)	0.013
Dominant model (CC vs. CG + GG)			1.405 (1.018–1.939)	0.038
Recessive model (CC + CG vs. GG)			1.107 (0.821–1.492)	0.506
HWE χ2	0.080	0.263		
HWE *p*	0.778	0.608		

*miRNA* microRNA; *MACE* major adverse cardiovascular events; *HR* Hazard ratio; *HWE* Hardy–Weinberg equilibrium, *CI* Confidence interval

### The G allele of miR-146a rs2910164 is susceptible to oxidation

**Fig. 5:**
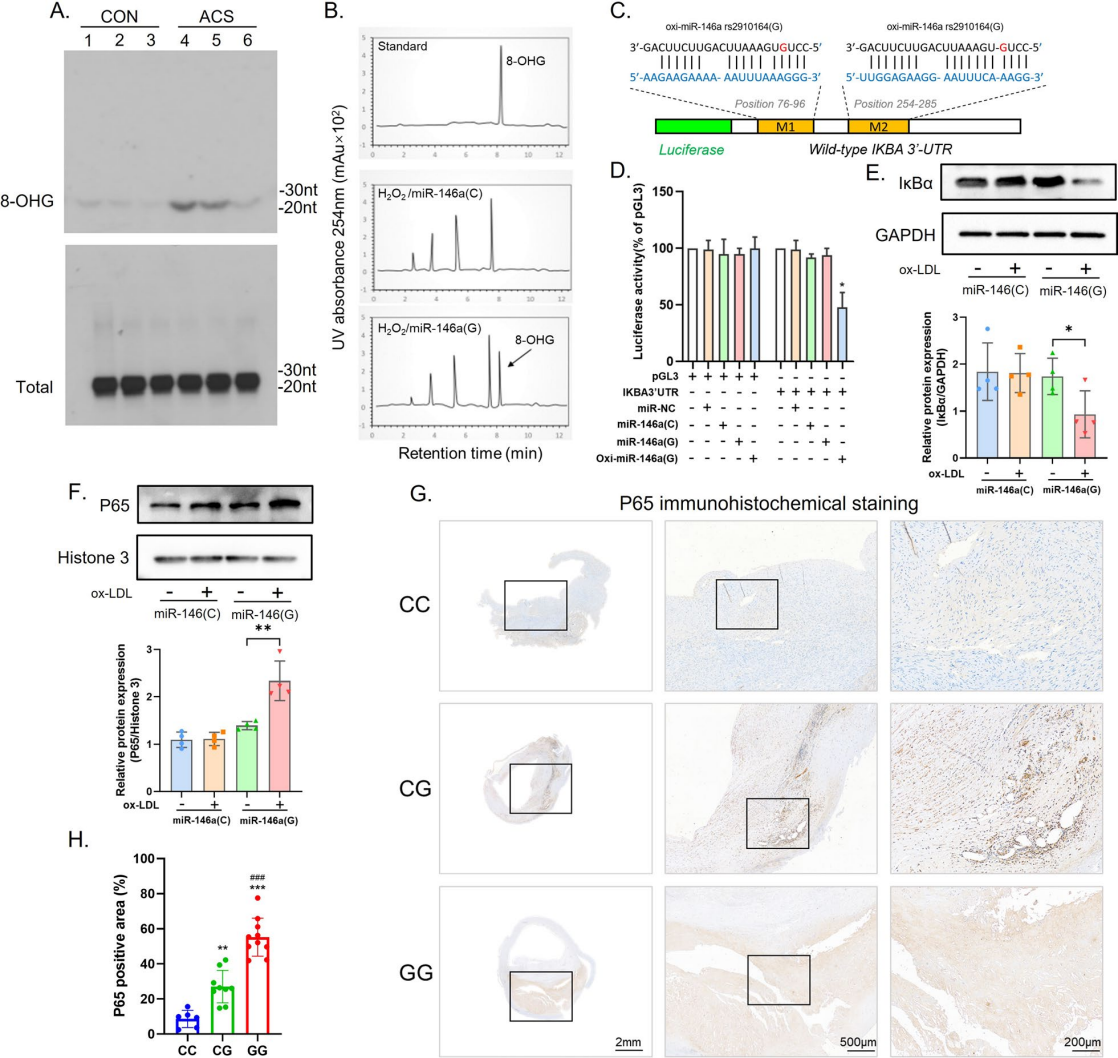
Oxidized miR-146a rs2910164 mismatches with the 3’UTRs of IKBA and activates the NF-κB inflammatory pathway. **A** MiRNA fractions were purified from monocytes isolated from controls and ACS patients, and 8-OHG levels were determined by Northwestern blotting using an anti-8-OHG antibody; **B** Analysis of 8-OHG in mir-146(C) and mir-146(G) by HPLC–UV spectroscopy; **C** Outline of luciferase reporter assay for validating the interaction of oxi-miR-146a(G) with IKBA 3′ UTR. The miRNA response elements (MREs) of oxi-miR-146a(G) in human IKBA 3′ UTR were predicted by the bioinformatics program RNAhybrid. The red color indicates the potential mismatching sites in which 8-OHG matches with A. M1, MRE1; M2, MRE2; **D** Oxidized miR-146a(G) reduces the luciferase activities of IKBA 3′ UTR. HEK293 cells were transfected with the luciferase constructs of the wild-type IKBA 3′ UTR, along with miR-NC, miR-146a(C), miR-146a(G), or oxi-146a(G). The empty vector pGL3 served as a control. ∗ p < 0.05 versus IKBA 3′UTR. The results are expressed as means ± SEM of at least three independent experiments; **E**–**F** THP-1 cells were transfected with miR-146a(C) and miR-146a(G), respectively, and then treated with ox-LDL or its control. IκBα(E) and P65 (F) protein levels were analyzed by immunoblot. Quantitative analysis of IκBα and P65 levels is shown in the lower panels. *p < 0.05 and ** p < 0.01 versus Control; **G**, **H** P65 immunostaining of atherosclerotic plaque sections of CC, CG, and GG genotypes. Left, overview of plaque; middle and right, higher magnification

Guanine can be hydroxylated by reactive oxygen species (ROS) to yield 8-oxo-7,8-dihydro-2'-deoxyguanosine (8OHdG) in DNA and 8-oxo-7,8-dihydroguanosine (8OHG) in RNA [19–21]. Wang, J.X et al. reported that miRNAs can also be oxidized in response to oxidative stress [22]. Oxidative stress plays a pivotal role in the process of atherosclerosis. Under such pathological conditions, ROS, including hydrogen peroxide (H2O2) and hydroxyl radicals (·OH), cause cell-damaging effects through oxidative modification of biomolecules such as proteins and nucleotides (DNA and RNA). Therefore, we infer that the G allele of miR-146a rs2910164 is susceptible to oxidation in ACS patients. To test whether the G allele can be oxidatively modified, we used an anti-8-OHG antibody that recognizes oxidatively modified 8-OHG. To avoid interference from other RNAs, miRNA fractions were purified from monocytes isolated from ACS patients and control individuals. As shown in Fig. 5A, we observed an increase in oxidatively modified miRNAs in monocytes from ACS patients. To verify whether the G allele of miR-146a rs2910164 could be oxidized to 8-OHG, we synthesized wild-type miR-146a(miR-146a(C)) and miR-146a mutated to G at rs2910164 (miR-146a(G)). They were then oxidized by the Fenton reaction and identified by HPLC–UV spectroscopy. Figure 5B shows that miR-146a(G) can be modified by oxidation. Together, these data suggest that miRNAs in ACS patients are more susceptible to oxidative modification, and miR-146a(G) can be oxidized to oxi-miR-146a(G).

### Oxidized miR-146a rs2910164 mismatches with the 3’UTR of IKBA and activates the NF-κB inflammatory pathway

8-OHG has been well characterized as pairing with A instead of C, resulting in changes in the targeting mRNA [23]. Through Targetscan, miRanda, miRMap, miTarBase and miRDB database predictions [24–28], it was found that miR-146a may interact with mRNAs related to the NF-κB signaling pathway (Additional file 1: Figure S3A, B, Additional file 3:  Excel2_miR 146a target). Besides, the above results suggest that patients carrying rs2910164 G allele have higher levels of inflammation. Therefore, we speculated that oxi-miR-146a(G) interact with mRNAs of the IKB protein family. After alignment, it was found that oxi-miR-146a(G) may be complementary to two sites in the 3'UTR of IKBA (Fig. 5C). To investigate whether these potential sites can be targeted by oxi-miR-146a(G), we made a luciferase construct of wild-type IKBA 3'UTR. The luciferase reporter assay indicated that oxi-miR-146a(G) caused a significant decrease in the luciferase activity of the IKBA 3' UTR, whereas miR-146a(C) and miR-146a(G) had no effect on the IKBA 3' UTR (Fig. 5D). Next, we transfected THP-1 with miR-146(C) and miR-146(G). In monocytes transfected with miR-146(G), a decrease in the protein expression level of IκBα and an increase in the protein expression of P65 were observed after ox-LDL treatment (Fig. 5E, F). This implies that ROS stimulated by ox-LDL oxidatively modified miR-146(G), which in turn inhibited the protein expression of IκBα, leading to an increased level of inflammation. Immunohistochemical staining of carotid plaques from atherosclerotic patients obtained after atherectomy showed that the expression of P65 protein was increased in the plaques of patients with CG and GG genotypes (Fig. 5G). This result further demonstrated that in patients with the rs2910164 G allele, miR-146a rs2910164 mismatches with the IKBA 3ʹUTRs and the intra-plaque NF-κB inflammatory pathways were activated.

## Discussion

This research includes a case–control study and a validation cohort. We first identified miR-146 rs2910164, but not miR-34b rs4938723, associated with the development of ACS using a case–control approach, and then followed with a validation cohort to determine a causal relationship between miR-146 rs2910164 and outcomes of post- PCI patient. The G allele of miR-146a rs2910164 was an independent predictor of MACE for ACS patients after PCI. The results provided new evidence for the relationship between miRNAs polymorphisms and both the onset of ACS and long-term prognosis of ACS patients.

The C allele of miR-146a rs2910164 has been reported to be a risk allele for nasopharyngeal carcinoma, while the G allele is a risk allele for asthma and vascular disease [29–31]. In our current case–control study, patients carrying the G allele of miR-146a rs2910164 had 1.270-fold (dominant model) or 1.402-fold (recessive model) higher risk of ACS than those with the C allele, indicating that the G allele of miR-146a rs2910164 is a susceptible allele for ACS. In addition, compared to C allele carriers, G allele carriers had a 1.405-fold(dominant model) higher incidence of MACE after PCI during the 5-year follow-up period. Moreover, the G allele of miR-146a rs2910164 was associated with serum inflammation factor levels.

Although several studies have shown that the miR-34 family is associated with the development of cardiovascular diseases such as coronary atherosclerosis and acute myocardial infarction [32–34]. However, our study revealed that polymorphism of miR-34b rs4938723 was not associated with the incidence of ACS and serum inflammatory factor levels.

Several articles have demonstrated that miR-146a inhibits inflammation levels by targeting the upstream NF-κB signaling proteins: interleukin-1 receptor-associated kinase 1 (IRAK1) and TNF receptor-associated factor 6 (TRAF6) through a negative feedback mechanism [35–37]. However, we observed that patients with miR-146a rs2910164 variant had higher levels of inflammation, so we speculate that perhaps the rs2910164 polymorphism alters the original target of miR146a and its physiological function.

Guanines in miRNAs can be hydroxylated by reactive oxygen species (ROS) to generate 8-oxo-7,8-dihydroguanosine (8OHG) [19–22]. 8-OHG pairs with A instead of C, resulting in changes in the miRNA-mRNA pairing [23].We first detected higher levels of miRNA oxidation, including miR146a, in monocytes from ACS patients. Secondly, it was demonstrated by HPLC–UV spectroscopy that mutated mir-146(miR-146a(G)), but not wild-type 146(miR-146a(C)), can be oxidatively modified. Oxi-miR-146a(G) activates the NF-κB pathway by mismatching with 3ʹUTR of IKBA. These results suggest that the pathogenicity of the miR-146a rs2910164 G allele is mediated in part by its mismatch with IKBA and its regulatory role in the inflammatory response. This conclusion is further supported by immunostaining for P65 in human atherosclerotic plaques.

Interestingly, some miRNAs were downregulated in CAD patients during our bioinformatics analysis. The downregulated genes are sorted according to the fold difference from large to small: miR-589, miR-520h, miR-144, miR-518f, miR-379, miR-512-5p, miR-668 and miR-554. Most of these miRNAs play roles in cancer, while there are few studies on them related to cardiovascular diseases.

MiR-520h can promote tumor growth and progression in hepatocellular carcinoma cell and breast cancer. Meanwhile, miR-520 h can target smad7/EMT, promoting the metastasis of bladder cancer cells [38]. Similarly, miR-518f-5p can reduce the expression of the tetraspanin CD9 in prostate cancer cells [39]. Danielle R Bond et al. also verified that the transfection of miR-518f-5p significantly decreased CD9 protein expression and increased breast cell migration in vitro [40]. These studies show that miR-518f-5p can enhance the migration and invasion ability of prostate cancer cells and breast cancer cells. As of miR-554, Yasuo Takashima et al. found that miR-554 is designated as a miRNA predictor in PCNSL (primary central nervous system lymphoma), which is associated with all categories such as Th-1/Th-2 and T-reg differentiation status, and stimulatory and inhibitory immune checkpoints [41]. MiR-554 has been identified as a reliable prognostic predictor of PCNSL.

However, miR-512-5p plays a significant inhibitory role in tumor progression. MiR-512-5p can target a variety of different genes, inhibit the expression of tumor related genes, and reduce the tumor cell’s ability of proliferation, migration, and invasion. Yixiang Huang et al. found that in breast cancer, circular RNA circRPPH1 acted as a miRNA sponge of miR-512-5p, which inhibits the combination of miR-512-5p and 3 '- UTR of STAT1, reduces the expression of STAT1, and promotes the he proliferation, migration and colony formation ability of Breast cancer cells [42].miR-668 plays a protective role against renal injury after ischemia–reperfusion (IRI). In ischemic acute renal injury, HIF1 induces miR-668 during the ischemic reperfusion (IRI) interval. This microRNA can inhibit the expression of mitochondrial protein 18 kDa (MTP18), maintaining mitochondrial dynamics and promoting the survival of renal tubular cells after IRI [43].

In acute myocardial infarction (AMI), miR-379 binds tumor necrosis factor-α-induced protein 8 (TNFAIP8) and inhibits its activity significantly, leading to the reduce of the level of apoptosis and the inhibition NK-κB signal pathway in H9c2 cells. This study shows that miR-379 can play a protective role in the heart [44].

MiR-144 can be used as a sequence specific inhibitor of ABCA1/G1 (ATP binding cassette transporter) mRNA to control gene expression. MiR-144 binds to the 3'UTR region site of ABCA1/G1.This will reduce the outflow of cholesterol to apolipoprotein A1 (ApoA1) mediated by the ABCA1/G1 of hepatocytes and macrophages, promoting inflammatory activation and atherosclerosis [45].

Studies have shown that COX2 promoter RNA contains two miR-589-5p binding sites. MiR-589 is an endogenous activator in the nucleus that can regulate the expression of COX-2 and PLA2G4A genes at the transcriptional level. Activation of the COX-2 gene by miR-589 requires AGO2 and GW182. COX-2 catalyzes the conversion of arachidonic acid and further metabolizes it to prostaglandins and other arachidonic acids. These arachidonic acids mediate many biological processes, including inflammation, cellular immunity, and cancer [46].

There are several limitations in our present study. Firstly, our data did not provide the expression levels of 3 different isoforms of miR-146a under the current circumstances. Secondly, the content of serum cytokines was measured at the time of admission and were not monitored dynamically. Thirdly, the validation cohort should be collected at different hospitals, or even multiple hospitals in different regions.

## Conclusion

The rs2910164 variant of miR-146a is closely associated with the risk of ACS in Chinese Han population. The patients carrying miR-146a with G allele inclines to be oxidized and may have a more serious pathological change and poorer post-PCI prognosis, partly due to the oxidatively modified miR-146a mispairing with 3’UTR of IKBA and activating NF-κB inflammatory pathways.

## Supplementary Information

**Additional file 1.** Supplementary material.

**Additional file 2.** Excel1_Different miRNA.

**Additional file 3.** Excel2_miR 146a target.

## Data Availability

The datasets used and analyzed during the current study are available from the corresponding author on reasonable request.

## Abbreviations

ACS: Acute coronary syndromes
PCI: Percutaneous coronary intervention
miRNAs: MicroRNAs
MACE: Major adverse cardiovascular events
MI: Myocardial infarction
SA: Stable angina
STEMI: ST-segment elevation myocardial infarction
NSTEMI: Non-ST-segment elevation myocardial infarction
UA: Unstable angina
CI: Confidence intervals
HR: Hazard ratio
OR: Odds ratio
CAD: Coronary artery disease
EAT: Epicardial adipose tissues
PCA: Principal component analysis
HWE: Hardy–Weinberg law of equilibrium
SNPs: Single nucleotide polymorphisms
8OHdG: 8-Oxo-7,8-dihydro-2'-deoxyguanosine
8OHG: 8-Oxo-7,8-dihydroguanosine
ROS: Reactive oxygen species
H2O2: hydrogen peroxide
·OH: Hydroxyl radical
IRAK1: Interleukin-1 receptor-associated kinase 1
TRAF6: TNF receptor-associated factor 6
BMI: Body mass index
SBP: Systolic blood pressure
DBP: Diastolic blood pressure
LVEF: Left ventricular ejection fraction
FBG: Fasting blood glucose
CK: Creatine kinase
CKMB: Creatine kinase isoenzymes
FIB: Fibrinogen
FDP: Fructose-1, 6-diphosphate
CRP: C-reactive protein
BNP: Brain natriuretic peptide
TC: Total cholesterol
TG: Triglyceride
HDL: High-density lipoprotein cholesterol
LDL: Low-density lipoprotein cholesterol
Apo: Apolipoprotein
Lpa: Lipoprotein a
HGB: Hemoglobin
WBC: White blood cell count
ACEI: Angiotensin converting enzyme inhibitor
ARB: Angiotensin receptor blocker
CCB: Calcium channel blocker
