# Supplementary material for: MiR-146a rs2910164 (G/C) polymorphism is associated with the development and prognosis of acute coronary syndromes: an observational study including case control and validation cohort
Source: J Transl Med. 2023 May 15;21:325. doi: 10.1186/s12967-023-04140-4 (PMC10186648; doi:10.1186/s12967-023-04140-4)
Supplement: Supplementary file 1 — Additional file 1. Supplementary material. [file 12967_2023_4140_MOESM1_ESM.docx]

**Additional methods:**

**Details of Genetic analysis:**

The genomic DNA was extracted from white blood cells using the commercially available DNA isolation kit (Tiangen Biotech, Beijing, China) according to the manufacturer’s instructions. Genotypes for individual DNA samples were genotyped using the ABI PRISM- SnapShot method (Applied Biosystem, CA, USA). In brief, the SnapShot reactions were performed in a 10 μL final volume containing primer mix (0.02-0.5 μmol/L), 5 μL of SnapShot Multiplex Ready Mix, and 4 μL of templates consisting of the multiplex PCR products. The multiplex PCR products had been purified with the Fermentas PCR Purification Kit (Fermentas, CA, USA) and the cycling program consisted of 25 cycles of 96 ℃ for 10 s, 51 ℃ for 5 s, and 60 ℃ for 30 s. Extension products were purified by incubation for 15min with shrimp alkaline phosphatase (1 U, Fermentas, CA) at 37 ℃ and a subsequent incubation for 15 min at 75 ℃ to denature the enzyme. After mixing the purified products (0.5 μL), Hidi-Formamide (8.5 μL) and 0.5 μL of GeneScanTM-120 LIZ Size Standard (Applied Biosystems), the final reaction mix was denatured at 95 ℃ 5 min. Genotypes of our 2 SNPs were identified by capillary electrophoresis (ABI PRISM3730 DNA Sequencer; Applied Biosystems). The results were analyzed with GeneMapper 3.0 software (Applied Biosystems). All SNapShot and PCR primers are listed in Table 1. Similar quality-control (QC) procedures were implemented for each assay. Genotyping quality control procedures for study have been described in detail (Song H, Ramus S J, Tyrer J, et al. A genome-wide association study identifies a new ovarian cancer susceptibility locus on 9p22. 2[J]. Nature genetics, 2009, 41(9): 996-1000). The genotype results were cross-validated by the blinded genotyping of 40 randomly selected samples. In order to evaluate the multiplex SNaPShot results, 40 samples were randomly selected and regenotyped by direct sequencing using a BigDye terminator (Applied Biosystem). All assays were 100% concordant. The following criteria was used as a measure of acceptable genotyping: (1) > 3% sample duplicates included; (2) concordance rate for the duplicates ≥ 98%; (3) overall call rate (by study) > 95%; (4) call rates > 90% for each individual plate and (5) no deviation from HWE in controls (*P*>=0.05).

**Northwestern University blot analysis**

Total RNA was extracted from cultured cells using TRIzol reagent. miRNA fractions of 18-24 nt were separated by polyacrylamide gel electrophoresis using the Total RNA Gel Purification miRNA Kit (Ambion) according to the manufacturer's instructions. The miRNA was isolated from the cells for HPLC analysis using the same method. Poly(A)+ mRNA was isolated using the Dynabeads mRNA Purification Kit (Ambion). purified miRNA samples were then separated on denatured 15% polyacrylamide gels and blotted on positively charged nylon membranes (Roche). Total RNA or purified mRNA samples were resolved in 1% agarose/formaldehyde gels and blotted on positively charged nylon membranes (Roche). After UV fixation, membranes are washed with PBS and then incubated in 1% BSA blocking solution for 1 hr at room temperature. The membranes were placed in oxidative damage marker monoclonal antibody (clone 15A3) (1:1,000; QED Bioscience) and incubated for 4 hours at room temperature. Membranes were then incubated with horseradish peroxidase-conjugated goat anti-mouse secondary antibody (1:1,200; Sigma). The signal was detected with Pierce ECL Western Blotting substrate (Pierce) and exposed on X-ray film (Kodak) according to the manufacturer's instructions.

**Oxidation of miRNA by the Fenton Reaction**

The synthesized miRNAs (80 μg) were incubated with 2 mM H2O2 by redox-cycling ferric iron (Fe3+, 0.5 mM) or cupric copper (Cu2+, 0.5 mM), respectively, with the reducing agent ascorbate (Asc, 5 mM) in 10 mM NaH2PO4/Na2HPO4 (pH 7.4). Samples were incubated at 37°C for 1 h, and the reactions were terminated by adding 10 mM of the metal chelators DFOM (for Fe3+) or cuprizone (for Cu2+) and placing the samples on ice. Nucleic acids were precipitated in 3 M ice-cold sodium acetate (pH 5.0) and 2.5 volumes of ethanol, centrifuged, washed with 70% V/V ethanol, and then hydrolyzed. The H2O2 concentration was determined spectrophotometrically at 240 nm (ε_240_ = 0.0394mM^-1^cm^-1^).

**Cell culture and transfection**

The human monocytic leukemia cell line, THP-1 cell was cultured in RPMI 1640 (Gibco Invitrogen, Paisley, UK), complemented with 10% fetal bovine serum (FBS) (Gibco) and 1% penicillin-streptomycin (Gibco). To induce monocytes differentiated into macrophages, PMA (Sigma-Aldrich) was used at a concentration of 25 ng/mL. Cells were incubated for 48 hours at 37°C and differentiation was visualized under an optical microscope. Transfection was carried out using Lipofectamine 3000.

**Luciferase assay**

The luciferase assay was performed as previously described (Lin et al., 2009) [21]. To generate reporter vectors with miR-146a or oxidized miR-146a binding sites, we cloned the entire 3'UTR of human IKBA into the pGL3 vector (Promega) immediately downstream of the luciferase gene stop codon. mutant and wild-type vectors for miRNA response elements were used as templates. For luciferase assays, cells in 24-well plates were cotransfected with 400 ng/well of the luciferase construct and 20 pmol of miR-146a, oxidized miR-146a with Lipofectamine 2000 (Invitrogen). The miR negative control (miR-NC) was used as a negative control. At 48 h post-transfection, cells were lysed and luciferase activity was measured using a luciferase assay system (Promega).

**Western blotting**

Use a solution containing 20 mM Tris-HCl (pH 7.4), 150 mM NaCl, 1% Nonider P-40, 0.5% sodium deoxycholate, 0.1% SDS, 0.004% sodium azide, 1% PMSF, 1% orthovanadate A solution of salt was used to lyse cells in RIPA buffer and 1% protease inhibitor cocktail at 4°C. Centrifuge the lysate at 10,000 G for 30 min at 4 °C to remove insoluble material. Collect the supernatant. Protein concentrations were measured by the bicinchoninic acid (BCA) protein assay. Equal amounts of protein (50 μg) from each sample were separated by 12% SDS-PAGE and transferred to nitrocellulose membranes. Membranes were blocked in 5% nonfat milk for 1 hr and then incubated with anti-IκBα (Proteintch), anti-P65 (Proteintch), anti-GAPDH (Proteintch), or Histone 3 (Proteintch) at 4°C. After four washes with PBS-Tween 20, horseradish peroxidase-conjugated secondary antibody was added. Signal was detected using Pierce® ECL Western Blotting Substrate (Pierce).

**Immunohistochemistry**

Briefly, sections were deparaffinized and endogenous peroxidase activity was blocked by incubating with 0.3% H2O2 in methanol for 30 min. After blocking with 3% normal bovine serum albumin, sections were incubated with primary antibody overnight at 4°C. Anti-P65 (Proteinch) antibody was applied at a 1:150 dilution.

**Table S1. Differently expressed miRNAs between atherosclerotic plaques and normal artery**

| miRNA | Site | FC | FC min | | FC max | | P Value | SNP ID | Allele | MAF＊ |
| --- | --- | --- | --- | --- | --- | --- | --- | --- | --- | --- |
| hsa-miR-21 | Carotis | 4.69 | | 3.23 | | 6.70 | 0.005 | -- | -- | -- |
|  | Femoralis | 10.67 | | 4.54 | | 16.83 | 0.001 |  |  |  |
|  | Aorta | 10.95 | | 6.25 | | 16.60 | <0.001 |  |  |  |
| hsa-miR-210 | Carotis | 4.13 | | 2.61 | | 7.23 | 0.004 | -- | -- | -- |
|  | Aorta | 5.97 | | 4.48 | | 10.15 | <0.001 |  |  |  |
| hsa-miR-34a | Carotis | 3.90 | | 3.22 | | 6.40 | 0.006 | -- | -- | -- |
|  | Femoralis | 4.69 | | 3.53 | | 6.36 | 0.002 |  |  |  |
|  | Aorta | 4.42 | | 3.48 | | 5.27 | 0.003 |  |  |  |
| hsa-miR-146a | Femoralis | 6.98 | | 5.64 | | 10.46 | <0.001 | rs2910164 | C>G | 0.384 |
|  | Aorta | 5.79 | | 1.84 | | 14.19 | 0.024 |  |  |  |
| hsa-miR-34b | Carotis | 3.29 | | 2.62 | | 5.13 | 0.004 | rs4938723 | T>C | 0.312 |
|  | Femoralis | 4.08 | | 2.60 | | 5.62 | 0.002 |  |  |  |
|  | Aorta | 4.07 | | 3.43 | | 4.44 | 0.001 |  |  |  |
| hsa-miR-155 | Femoralis | 3.25 | | 2.47 | | 4.91 | 0.002 | -- | -- | -- |
|  | Aorta | 4.18 | | 2.56 | | 5.68 | 0.001 |  |  |  |
| hsa-miR-1308 | Femoralis | 3.65 | | 2.84 | | 4.46 | 0.002 | -- | -- | -- |
|  | Aorta | 3.71 | | 2.11 | | 7.66 | 0.018 |  |  |  |

Abbreviations: FC = fold change, MAF = Minor Allele Frequency; ＊：MAF in East Asian

**Table S2. SnapShot and PCR primers of 2 SNPs**

| Gene SNP Forward primer Reverse primer SNaPShot primer |
| --- |
| miR-146a rs2910164 AGCCGATGTGTATCCTCAGC TGCCTTCTGTCTCCAGTCTTC ttttttttttttttttttttttttttGGTTGTGTCAGTGTCAGACCT  miR-34b rs4938723 TCTCACCTCCTCTGGGAACC GCCAGGCAGCTTGTTAGTTAC ttttttttttttttttttttttGGGAACCTTCTTTGACCTAT |

Abbreviations: SNP: single nucleotide polymorphisms; miRNA: microRNA.


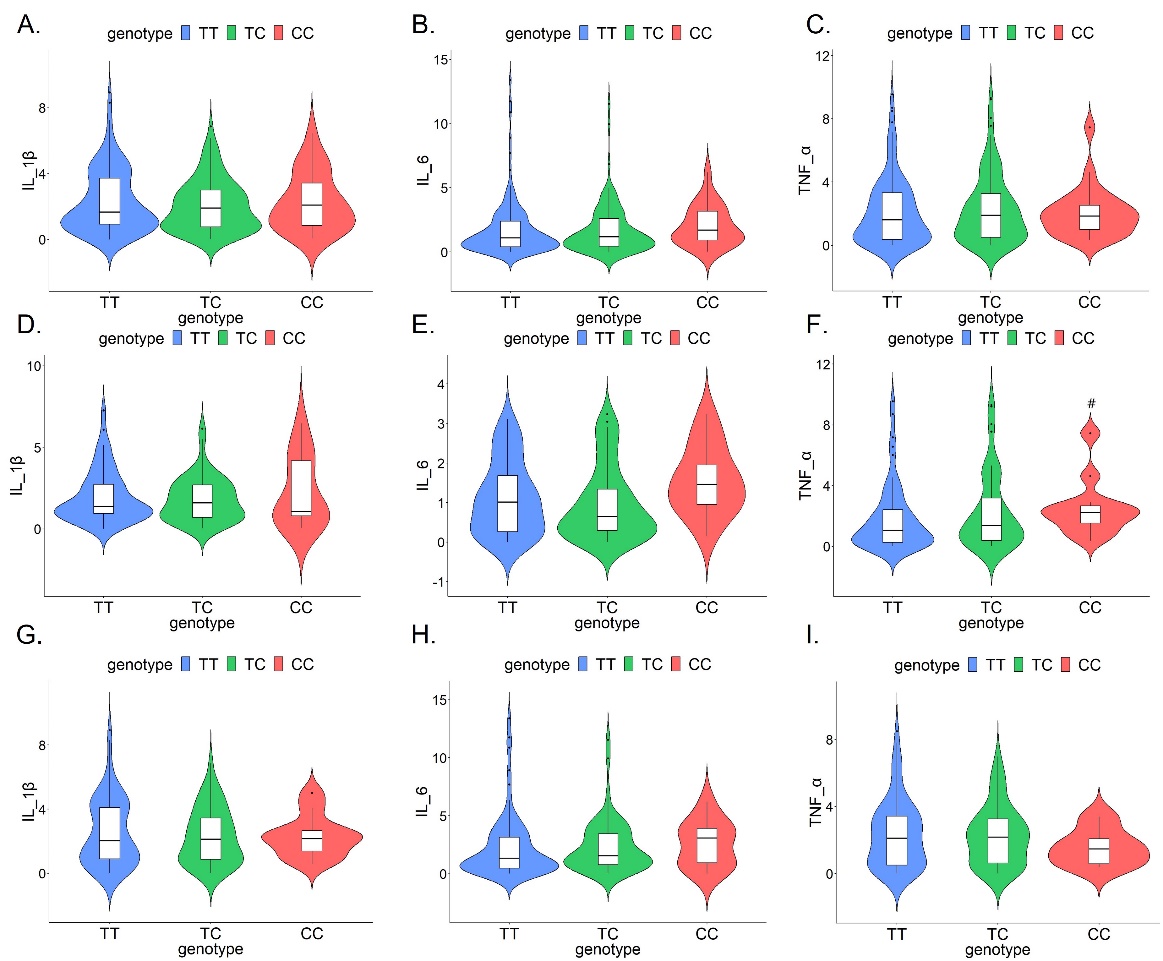
FigureS1.


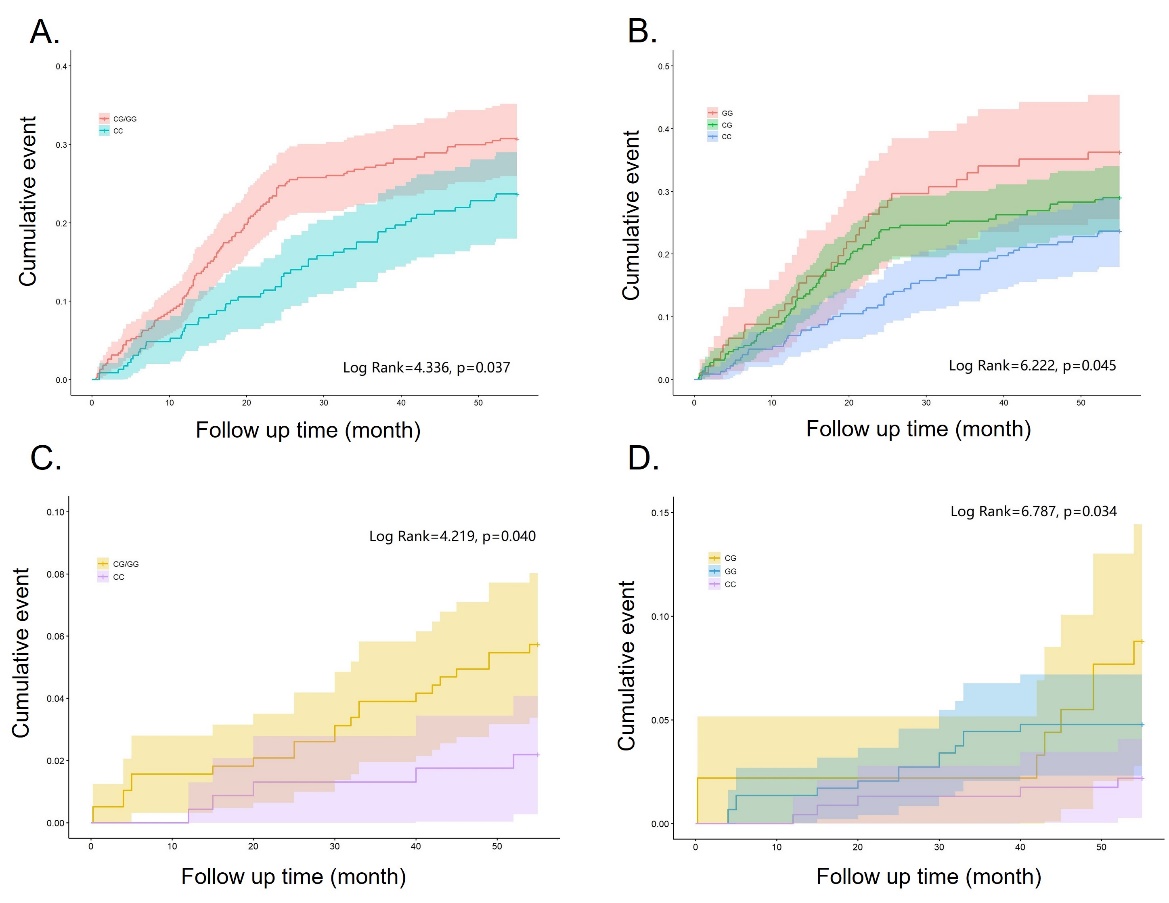
FigureS2.

FigureS3.
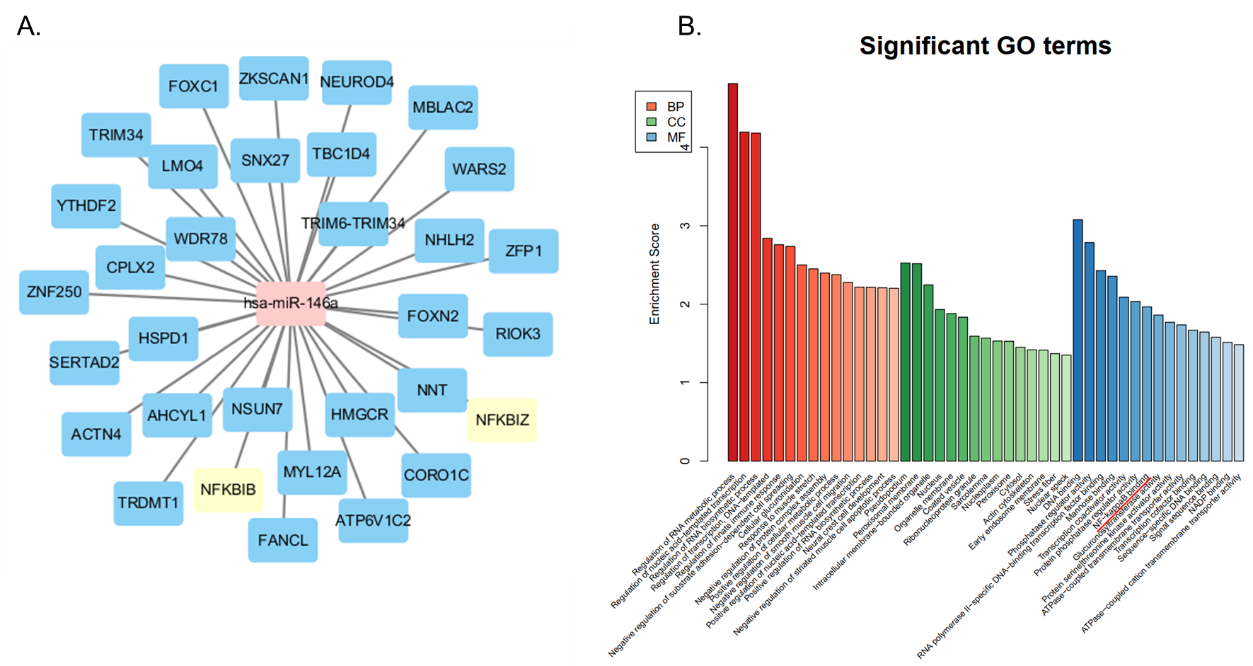


FigureS4.
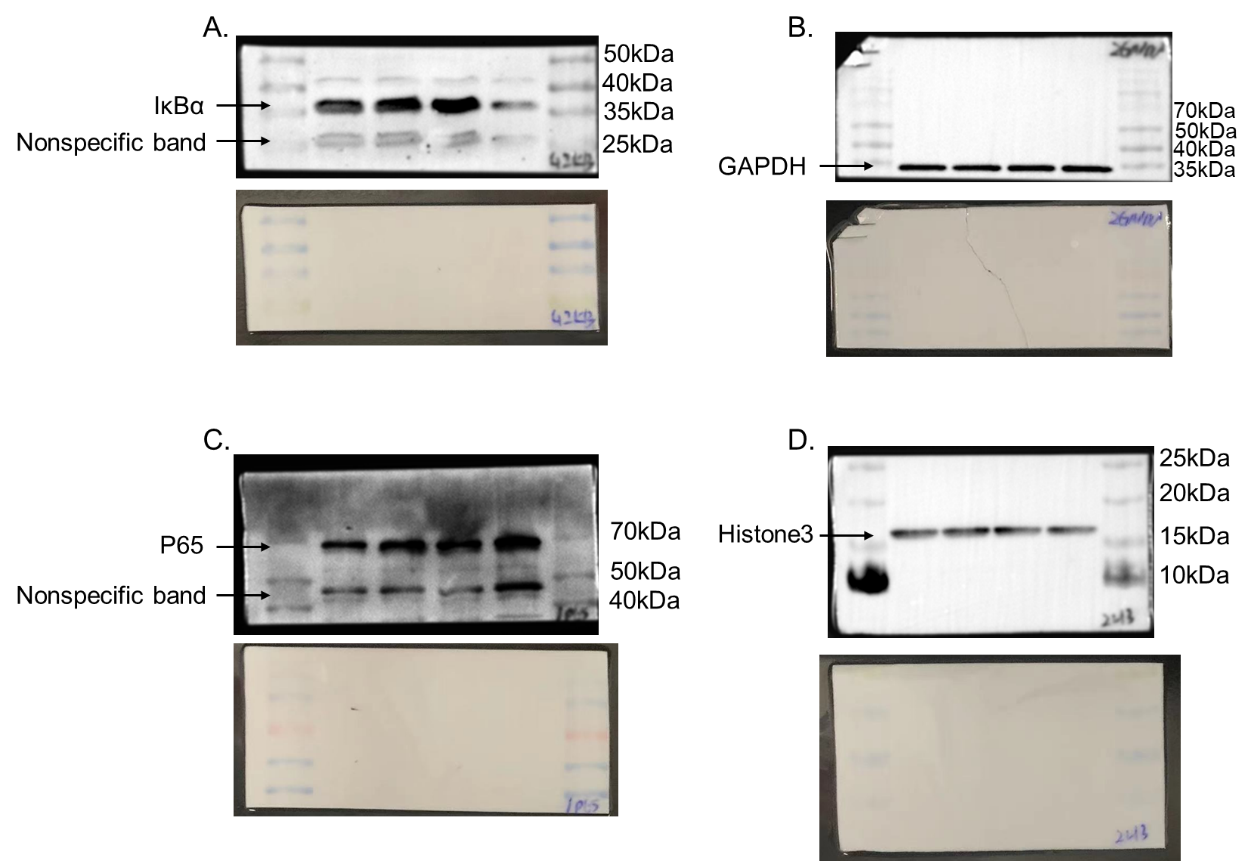


**Figure legend:**

FigureS1. MiR-34b rs4938723 polymorphism is not associated with inflammatory factor levels

(A-C). Among all 1171 individuals, serum IL-1β, IL-6 and TNF-α levels in different genotypes of miR-34b rs4938723. The unit is pg/ml;

(D-F). Within control group, serum IL-1β, IL-6 and TNF-α levels in different genotypes of miR-34b rs4938723. The unit is pg/ml;

(G-I). Within ACS group, serum IL-1β, IL-6 and TNF-α levels in different genotypes of miR-34b rs4938723. The unit is pg/ml.

FigureS2. Kaplan-Meier cumulative event rate curve of clinical outcomes of post-PCI patients.

(A-B). Kaplan-Meier cumulative event rate curve for MACE judging by miR-146a rs2910164 polymorphism. MACE: major adverse cardiovascular events;

(C-D). Kaplan-Meier cumulative event rate curve for death judging by miR-146a rs2910164 polymorphism.

FigureS3. Prediction and functional analysis of miR-146a target genes.

(A). miRNA-mRNA relationship pairs were predicted by Targetscan, miRanda, miRMap, miTarBase, and miRDB databases, and network graph visualization was performed using cytoscape 3.4.0 software;

(B). Functional GO annotation was performed on the predicted target genes. The red line marks the NF pathway. BP: Biological Process, MF: Molecular Function, CC: Cellular Component.

Figure S 4. Raw images of immunoblotting.

(A). IκBα immunoblotting; (B). GAPDH immunoblotting; (C). P65 immunoblotting; (D). Histone3 immunoblotting.
